# Field evaluation and automation of a method for the simultaneous determination of nitrogen oxides, aldehydes and ketones in air

**DOI:** 10.1155/S1463924696000090

**Published:** 1996

**Authors:** Andreas H. J. Grömping, Karl Cammann

**Affiliations:** 1 Lehrstul für Analytische Chemie Anorganisch-Chemisches Institut Westfälische- Wilhelms-Universität Münster Münster D-48148 Germany; 2 Institut für Angewandte Physikalische Chemie Forschungszentrum Jülich GmbH Jülich D-52425 Germany


Journal of Automatic Chemistry, Vol. 18, No. 3 (May-June 1996), pp. 121-126

### Field evaluation and automation of a method for the simultaneous determination of nitrogen oxides, aldehydes and ketones in air

**Andreas H. J. Grömping* and Karl Cammann**

*Lehrstul für Analytische Chemie, Anorganisch-Chemisches Institut, Westfälische-Wilhelms-Universität Münster, D-48148 Münster, Germany*

*The automation of a method for the simultaneous determination of nitrogen oxides, aldehydes and ketones is explained, and its applicability is shown by means of several field tests. All components were sampled together using impingers filled with a solution of 2,4-dinitrophenylhydrazine (DNPH). The corresponding derivatives were separated by HPLC and quantified by UV/Vis-absorbance at 345 nm. Measurements were also performed with solid sorbents. In order to explore the potential of this recently developed method, field samples were taken from: exhaust from automobiles, gas stoves, candle and cigarette smoke, and ambient air from restaurants. The variety of these sources demonstrates the versatility of this technique. The new method was validated in these field measurements for $NO_2$, NO, formaldehyde, acetaldehyde, acroleine, and acetone in the range of 20–100 000 ppbv, 50–100 000 ppbv, 10–15 000 ppbv, 20–250 ppbv, 10–150 ppbv, and 1–25 ppbv, respectively.*

### Introduction

Because of their frequent occurrence, nitrogen oxides, aldehydes, and ketones are important environmental pollutants [1, 2]. Since almost everyone is frequently exposed to formaldehyde and/or nitrogen oxides, the risks they pose to health cannot be neglected [3]. For some years formaldehyde has been discussed as a potential carcinogen [4]. Many acute dangers of formaldehyde are well known. For example concentrations above 30 ppmv exposure cause oedema of the lungs, which may be fatal [3]. Besides, formaldehyde is also an important contact dermatide [5].

While formaldehyde is a major chemical product worldwide [1], nitrogen oxides appear principally as byproducts and intermediates only. Both formaldehyde and nitrogen oxides are formed in combustion reactions [6]. Therefore, there are several cases in which formaldehyde (together with other aldehydes and ketones) and nitrogen oxides occur together.

There are a number of methods for the determination of formaldehyde and nitrogen oxides: for example, the classical methods for the analysis of formaldehyde, which are based on colorimetric techniques, are the 3-methyl-2-benzthialonhydrazine-(MBTH) procedure [7], the chromotropic acid-(CTA) [8], and the pararosaniline-(PRA) method [9]. Since some of these methods suffer from interferences, other methods using chromatographic separations have been proposed [10, 11]. In recent years the DNPH-method has become the most important method for the analysis of formaldehyde [12–16].

For the determination of nitrogen oxides, the Saltzman method [17] or the triethanolamine method [18] have been applied, which are also based on colorimetric techniques. But for the analysis of formaldehyde and nitrogen oxides, two separate methods were necessary [19, 20]. For this reason a new method for a simultaneous determination of aldehydes, ketones, and nitrogen oxides was developed [21, 22]. This paper demonstrates the versatility of this new method with the results of several field tests.

The various methods described above are almost exclusively used for single measurements. Nevertheless, an automated continuous control would be preferable. Therefore, a wet chemical analyser for the analysis of formaldehyde was developed by Monsen and Stock, which is based on the PRA method [23]. However, the PRA technique is prone to interference from other aldehydes and ketones. This paper describes the automation of the simultaneous determination of formaldehyde (together with other aldehydes and ketones) and nitrogen oxides [24].

### Experimental

### *Reagents*

Formaldehyde (37%), acetaldehyde, acetone, acrolein, propionaldehyde, methanol, hydrochloric, and phosphoric acid were Merck analytical grade. Deionized, twice distilled water, and acetonitrile (liquid chromatography grade, Merck) were used as eluents for HPLC. To reduce the blank, 2,4-dinitrophenylhydrazine purchased from Riedel-de-Haen was recrystallized twice from acetonitrile.

### *Apparatus*

As portable sampling pumps, Models S 2500 and Alpha 1 from E.I. du Pont de Nemours & Co., Inc, and the Quantimeter 1000, from Dräger were used. The HPLC system consisted of an Altek Model 100 A pump or a Knauer HPLC pump, a Rheodyne Model 7125 injection valve or a Rheodyne Model 7126 pneumatic injection valve both with a 20 µl loop. As detectors a Zeiss Model PMQ 3 UV-visible absorbance detector with an Uvicon 6 µl cell or a Knauer UV-visible absorbance detector were used. A Machery & Nagel Poligosil $C_{18}$ (5 µm, 20 × 4 mm)

* *Present address: Institut für Angewandte Physikalische Chemie, Forschungszentrum Jülich GmbH, D-52425 Jülich, Germany.*

0142-0453/96 $12.00 © 1996 Taylor & Francis Ltd.

guard column was used with a Machery & Nagel Poligosil $C_{18}$ (5 μm, 250 × 4 mm) analytical column.

A Spark Timer PT 740 was used for the automation. To measure mainstream cigarette smoke according to the Deutsche Industrie Norm (DIN [24]), a Borgwaldt smoke machine was used (kindly loaned by the Fraunhofer Institut für Luft- und Aerosolforschung, Hannover). Analysis of side-stream cigarette smoke was done at the same institute by sampling in a polypropylene box of 1200 l (0·8 m × 0·8 m × 1·9 m).

### Air sampling

Several investigations were carried out using the impinger technique. The impingers were filled with a solution of 1·5 mg DNPH in 2.·5 ml acetonitrile acidified with 100 μl 1 N hydrochloric acid. An air stream of 0·8 l/min was drawn through the impinger by a personal sampling pump. To protect the pump from the evaporating voltatile solvents, a trap filled with methanol/dry ice was used. Aldehydes and ketones react with DNPH in the impinger to produce the corresponding hydrazones. Nitrogen dioxide reacts to form DNPazide. Nitric oxide was determined by oxidation to nitrogen dioxide by means of potassium dichromate. For measurement of the sum of the nitrogen oxides, a tube, which was placed before the impinger, was filled with 200 mg $K_2Cr_2O_7$, which had been acidified with two to three drops of concentrated phosphosphoric acid. If this oxidation layer is placed before the impinger, aldehydes and ketones cannot be analysed, because $K_2Cr_2O_7$ oxidizes alcohols to aldehydes or ketones.

Sampling with solid sorbents with acidified DNPH was also used. Coating of Chromosorb P with DNPH, preparation of sampling tubes and desorption was done as described in another paper [22]. In each case, 20 μl of the absorbing solution was analysed by HPLC after sampling.

### Automation

The automation was accomplished using the apparatus shown in figure 1. The central unit is the timer (5), which opens and closes the solenoid valves (2) and the pneumatic HPLC valve (7) through the controlling units (6 and 12). The timer also starts and stops the personal air sampling pump (14). Each determination begins with the filling of the impinger (4) up to a certain level (usually 2 ml). This level is controlled by a photoelectric beam (3) using a light diode (17) and a photo transistor (16); it can be adjusted with a screw (18). The next step is to draw air through the impinger by means of the air sampling pump (14), which is protected from evaporizing solvents using a methanol/dry ice trap (13). The evaporated solvents are replaced and the solution is mixed by a brief switching on of the air sampling pump. Then the solution is pumped through the HPLC-loop (7) via a peristaltic pump (11), thereby rinsing and filling the loop. Finally, the content of the loop is injected onto the HPLC-column and analysed by the HPLC/UV system (9).

The possibilities of this automated method were tested by the sampling of candle smoke with the impinger technique.

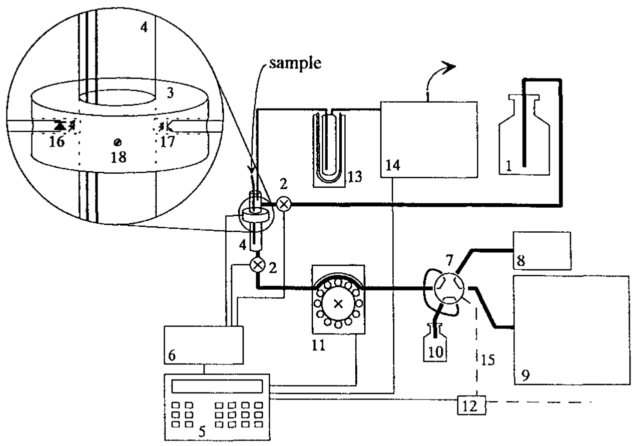

*Figure 1. Apparatus for the quasi-continuous, automated determination (reproduced from reference [24] with permission). Where 1: reservoir with absorber solution, : Teflon solenoid valves, 3: photoelectric beam, 4: impinger, 5: timer, 6: controlling unit for 2 and 3, 7: pneumatic HPLC-valve with loop, 8: HPLC pump, 9: HPLC/UV-apparatus, 10: waste, 11: peristaltic pump, 12: controlling unit for HPLC valve, 13: methanol/dry ice cooling trap, 14: personal air sampling pump, 15: compressed air supply, 16: light diode, 17: photo transistor, 18: adjustment screw for photoelectric beam.*

This sampling was carried out using a funnel ($r$ = 5 cm), placed upside down above the flame with the rim at the same height as the tip of the flame. It was then connected via a PVC tube to the impinger [22]. Drawing air with a stream of 0·8 l/min (much more than the gas developing from the burning flame) allowed a complete sampling of the candle smoke.

### Results and discussion

### Automation

The use of the apparatus described above has some advantages compared with the simple manual injections. Since it repeats the same process over and over again the measurements are very reproducible. This resulted in an improved correlation coefficient: 0·999 instead of 0·994 for the manual technique.

Figure 2 shows the results of 10 measurements—these were done every 10 min over a period of 100 min. Small shifts were observed (10 ppmv and 50 ppbv for nitrogen dioxide and formaldehyde, respectively), which were due to shifts in the magnitude of the flame. This test, despite the small variations, demonstrates the applicability of the automated method for field experiments. Nevertheless, the manual technique is still helpful for analysing single samples where a quasi-continuous determination is unnecessary.

### Gas stoves

Gas stoves were examined to compare the new method with other procedures. Air sampling was carried out with impingers filled with deionized water. Using a personal sampling pump, a flow rate of 0·2 l/min was drawn through the impinger. The absorber solution was subsequently analysed using the following methods:

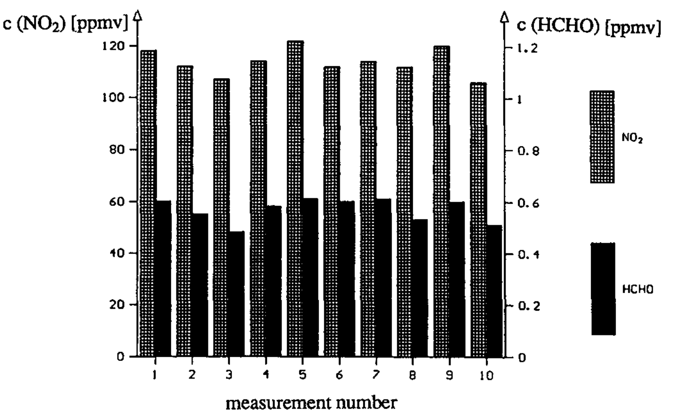

*Figure 2. Repeated measurements of the emission of formaldehyde and nitrogen dioxide of a candle (for chromatographic details see figure 5; reproduced from reference [24] with permission).*

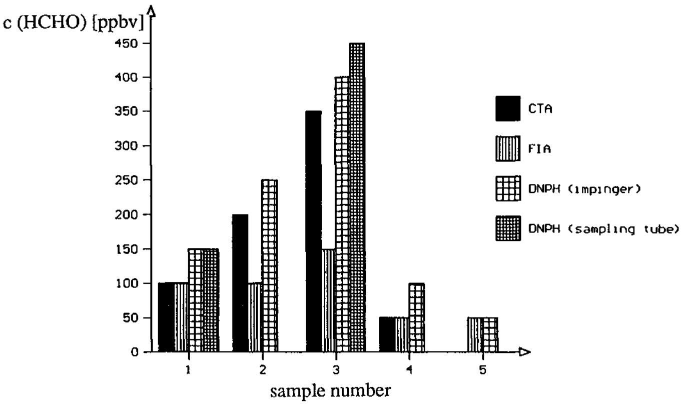

*Figure 3. HCHO-concentration of two gas stoves, measured with different methods.*

(1) CTA procedure using the method described by Geisling *et al.* [8].
(2) DNPH method (0·5 ml of the absorber solution were added to 2 ml of a solution of 1 mg DNPH in 2 ml acetonitrile, acidified with 15 µl 1 N hydrochloric acid).
(3) Enzymatic FIA procedure [26].

Samples were taken simultaneously with sampling tubes; 30 piston strokes of 0·1 l were drawn through each sampling tube. Figure 3 shows a comparison of the concentrations as measured with different methods. (In the second sample, a sampling tube with an oxidation layer was used for the measurement of the sum of nitrogen oxides. Therefore, formaldehyde could not be determined in this case.)

As the enzymatic FIA method was to be compared with the DNPH method, only a very low volume of air was drawn through the impinger and sampling tube. For this reason, the deviation of the data was unusually high at 50 ppbv. Considering this deviation, the data showed good agreement. The measurement with the enzymatic FIA method of the second and third sample was the only one to deviate substantially from the other data. The concentrations even in the worst case were below the German maximum working place concentration (MAK) of 0·5 ppmv; however, they were slightly above the 0·1 ppmv value, which is recommended by the German Bundesgesundheitsamt (BGA) for indoor air. The determined concentrations were similar to those measured by Matthews and Howell: 35–400 gm/m$^3$ (30–350 ppbv) [27].

The nitrogen oxide concentrations were measured simultaneously using the new method. For the first sample, 300 ppbv nitrogen dioxide were measured with both sampling methods, the tube and the impinger. In the second sample, the sum of nitrous oxides was found to be 500 ppbv with the sampling tube. The nitrogen dioxide concentration was 400 ppbv. Because of the low sample volume (see above), the deviation was a very high 100 ppbv. Therefore, the concentration of NO could only be estimated from the difference of these two measurements to be about 100 ppbv. In the third measurement, 700 and 600 ppbv were measured with the sampling tube and the impinger, respectively. Samples 4 and 5 were analysed with the impinger technique only. The nitrogen dioxide concentrations were 200 and 100 ppbv, respectively.

Despite these problems, which can be avoided simply (for the DNPH method) by taking greater sample volumes, the new method was able to determine nitrogen oxides and formaldehyde simultaneously. All nitrogen oxide concentrations were well below the MAK value of 5 ppmv and they were similar to those measured by Matthews and Howell [27]; 200–2400 g/m$^3$ (100–1200 ppbv). The concentrations closely matched comparable samples.

*Automobile exhaust*

In outdoor air, automobile exhaust is by far the most important source of formaldehyde and nitrogen oxides. So exhaust from automobiles was a useful test for the new method. Nine automobiles were used and measurements were carried out with solid sorbent tubes. The tubes were inserted 10 cm into each exhaust pipe; the exhaust was drawn for ten minutes through the tubes at 0·8 l/m. Because of the high nitrogen oxide concentration in diesel engine exhaust, the samples from the second car (which had a diesel engine) were taken for two and five minutes (third sample) only. The exhaust of each car was examined three times: the first measurements were carried out when the engines were still cold with the normal sampling tube. For the subsequent second measurement the tubes were filled with an oxidation layer in addition to the sampling layer for the sum of nitrogen oxides as described above. The third samples were taken with normal sampling tubes when the engines were warm.

Because the oxidation layers consisted of potassium dichromate, which oxidizes alcohols to aldehydes or ketones, the second measurements could not be used for the determination of aldehydes and ketones. For the determination of nitric oxide the mean value of the first and the third measurement for nitrogen dioxide was substracted from the second one. The concentration of NO was generally much higher than that of $NO_2$. Only the diesel engine emitted great quantities of nitrogen dioxide 130 ($\pm$20) ppmv as well. For this reason the NO concentration could only be estimated to be 100 ($\pm$50) ppm.

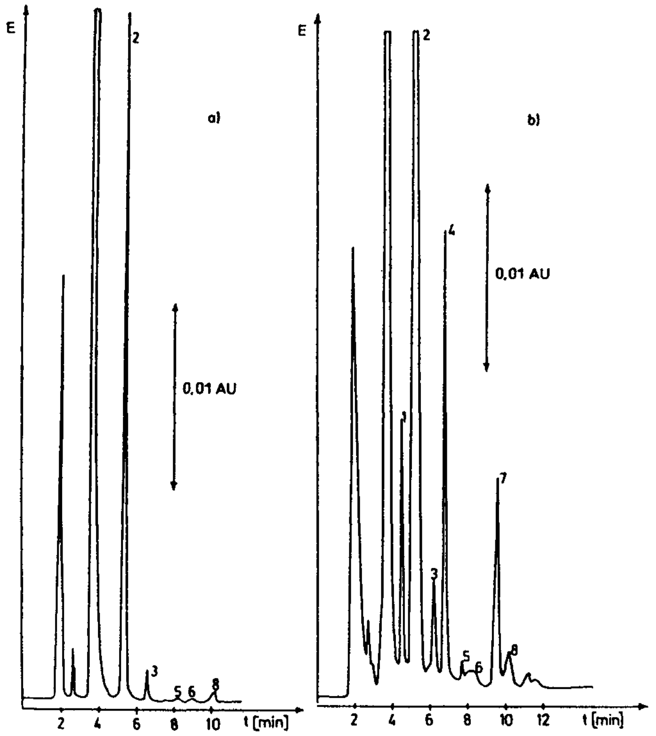

*Figure 4. Chromatogram of automobile exhaust (a) without and (b) with oxidation layer. Column: Polygosil $C_{18}$ (5 μm, 250 × 4 mm); flow rate: 1·0 ml/min; eluent: 60% acetonitrile/40% water; wavelength: 345 nm; injection volume: 20 μl; peak identification: 1: DNPazide; 2: formaldehyde-DNPhydrazone; 3: acetaldehyde-DNPhydrazone; 4: unknown; 5: acetone-DNP-hydrazone; 6: propanal-DNPhydrazone; 7, 8: unknown. (The first peaks belong to the derivatizing agent and are not indicated for this reason.)*

*Table 1. Comparison of the data [ppbv] obtained in this work for exhaust measurements with data from the academic literature.*

| Reference | [28] | [29] | [30] | This work |
|---|---|---|---|---|
| Formaldehyde | 1·85 | 0·47–0·7 | 2·9–32 | 0·64–15 |
| Acetaldehyde | 0·39 | 0·09–0·18 | 1·7–8 | 0·07–0·66 |
| Acrolein | 0·06 | 0·05–0·09 | 1·7–3·8 | 0·04–1·6 |
| Acetone | — | — | See acrolein | 0·06–0·24 |
| $NO_2$ | — | — | 15–3900 | 60–195 |
| NO | — | — | See $NO_2$ | 0·3–5·5 |

Figure 4 shows the difference between sampling with and without the oxidation layer. Peak 1, which corresponds to DNPazide, appears only in the second chromatogram. It can be concluded that there was almost no nitrogen dioxide but a considerable amount of NO in the exhaust. Other peaks, such as that of formaldehyde-DNPhydrazone, were seen to grow—this was due to the oxidation of alcohols (for example methanol) to the corresponding aldehydes or ketones.

Table 1 shows that the data obtained with this new method were in the same range as data published previously by other authors, showing the reliability of this method. (Only one study measured aldehydes, ketones, and nitrogen dioxide [30], but this does not determine nitric oxide and gives only the sum of acetone and acrolein.)

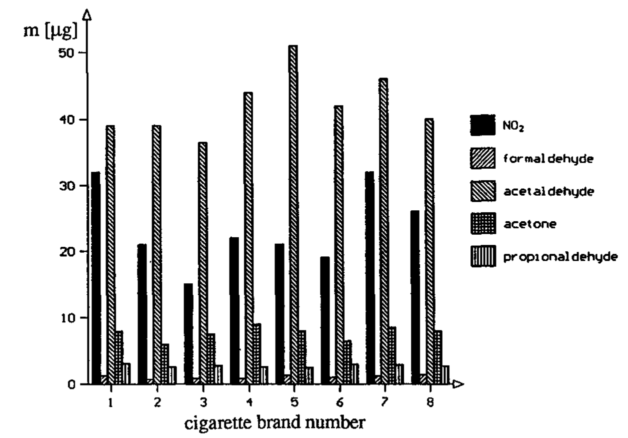

*Figure 5. Mean values for different cigarette brands (data from four measurements, each related to one cigarette).*

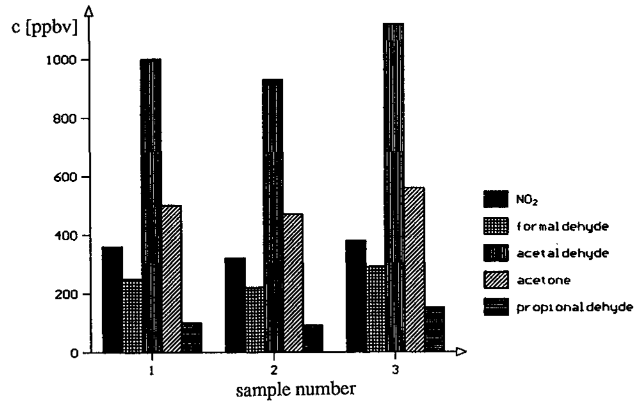

*Figure 6. Different measurements of concentrations in the side-stream of cigarettes (data related to one cigarette; 1000 ppbv in a volume of 1·2 $m^3$ correspond to 2·46 mg nitrogen dioxide, 1·61 mg formaldehyde, 2·25 mg acetaldehyde, and 2·89 mg propionaldehyde and acetone).*

*Cigarette smoke*

For the measurement of mainstream cigarette smoke, the cigarettes were connected to sampling tubes. By means of the smoke machine, filter cigarettes of 83 mm length were smoked, taking a 35 ml puff of two seconds duration each minute, to a 30 mm butt length. Four cigarettes of eight brands were examined and figure 5 shows the mean values.

The determination of side-stream cigarette smoke was carried out by smoking three filter cigarettes one after the other in a box. The mainstream was drawn out of the box. The air from the box was analysed by drawing 0·8 l/min for 15 minutes through a sampling tube. The concentrations of the components measured in this work were much higher in the side-stream than those of the mainstream as a comparison of figure 5 (mainstream) and figure 6 (side-stream) demonstrates. In the mainstream only small amounts of formaldehyde were found, whereas the acetaldehyde concentrations were high. In the side-stream, the ratio of formaldehyde to acetaldehyde was significantly higher than in the mainstream.

*Table 2. Survey of the mean values [ppbv] from different sources.*

| | $NO_2$ | NO | HCHO | $CH_3CHO$ | acrolein | acetone |
|---|---|---|---|---|---|---|
| Gas-stoves | 500 | 100 | 200 | n.d. | n.d. | n.d. |
| Cigarettes | 20 | — | 10 | 20 | 10 | 1 |
| Restaurants | 500 | — | 250 | n.d. | n.d. | n.d. |
| Candles | 10 | n.d. | 0·05 | 0·06 | 0·05 | n.d. |
| Fire places | 100 | 50 | 200 | n.d. | n.d. | n.d. |
| Chimney | 1·000 | — | 250 | n.d. | n.d. | n.d. |
| Automobile exhaust | | | | | | |
| —gasoline engine | <200 | 2·500 | 6·000 | 250 | 150 | 25 |
| —diesel engine | $10^5$ | $10^5$ | 5·000 | n.d. | n.d. | n.d. |

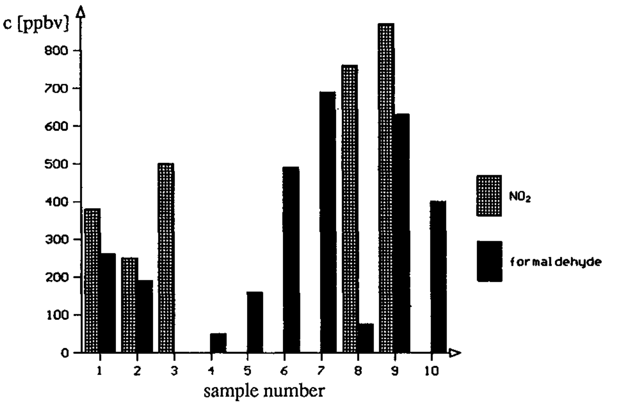

*Figure 7. Measurements of air in different restaurants.*

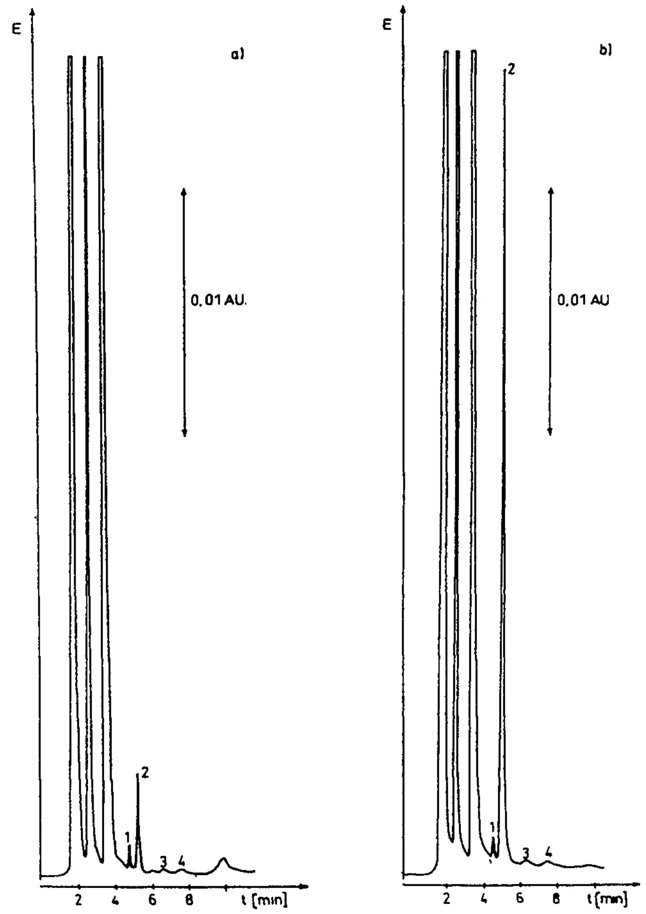

*Figure 8. Chromatogram of the measurements in restaurant V, (a) before smoking (measurement 8) and (b) during smoking (measurement 9). For chromatographic details see figure 4; peak identification: 1: PNDazide; 2: formaldehyde-DNPhydrazone; 3: acetaldehyde-DNPhydrazone; 4: acetone-DNPhydrazone. (The first peaks belong to the derivatizing agent and are not indicated for this reason.)*

### *Restaurants*

To demonstrate the possibilities of the new method for the analysis of nitrogen oxides and formaldehyde in our every day social environment, the air in six restaurants was analysed. In the first and in the last restaurant only one measurement was made, while two were made in the others. (Samples 2 and 3 were taken in the second; 4 and 5 in the third; 6 and 7 in the fourth, and 8 and 9 in the fifth restaurant.) Measurements were carried out by means of sampling tubes. Air was drawn for 15 minutes with a sampling rate of 0·8 l/min through the sampling tubes.

Considerable differences in formaldehyde concentrations, depending on the amount of smoking were noticed (see figure 7). There were no smokers in the third restaurant during the first measurement (measurement No. 4). During the second one (No. 5) somebody started smoking at the neighbouring table. The difference between the two measurements in the fifth restaurant (samples 8 and 9) is striking. During the first measurement almost nobody was smoking, while several people started smoking during the second measurement, resulting in a significant rise of the formaldehyde-DNPhydrazone peak in figure 8. The concentrations of acetaldehyde, acetone and nitrogen dioxide were almost constant. Apart from measurements 7 and 9, all concentrations were below the German maximum working place concentration (MAK) of 0·5 ppmv, but some were above the 0·1 ppmv, which is recommended by the German Bundesgesundheitsamt (BGA) for indoor air.

### *Range of concentrations*

The reported measurements provide some data for discussion of the possibilities of the new simultaneous method. Some conversions are necessary, however, to allow a comparison of the data from the different sources. The emissions of candles and cigarettes had to be converted into reasonable indoor air concentrations. For the candles, the data were applied to a room of 30 $m^3$ and a burning-time of one hour. The mainstream emission of cigarettes was applied as well to a room of 30 $m^3$ for smoking of one cigarette. The mean values given in table 2 were obtained using these conversions.

Despite the large range of concentrations, all could be measured with the method presented using suitable preconcentrations. For example all gases emitted from a candle were sampled, while only a small part of the automobile exhaust was drawn through the sampling tubes. Following this method, a determination of formaldehyde concentractions, differing by five decades, was possible.

### Acknowledgement

Financial support of the Bundesministerium für Forschung und Technologie (AZ.: 325-4007-07INR223) is gratefully acknowledged.

### References

1. Forth, W., Henschler, D. and Rummel, W., *Allgemeine und spezielle Pharmakologie und Toxikologie*, 5th edition (Wissenschaftsverlag Zürich, 1987).
2. Bundesansalt für Arbeitsschutz; *Formaldehyd—Verwendung, Gefahren, Schutzmaßnahmen;* 4th edition (Dortmund, 1985).
3. Feinauer, G., *GIT Fachzeitschrift Labor*, **11** (1985), 1111–1121.
4. Fleig, I. M., Petri, N., Stocker, W. G. and Thiess, A. M., *Journal of Occupational Medicine*, **24** (1982), 1009–1012.
5. Heilman, B., *Environment Science, and Technology*, **16** (1982), 543A–547A.
6. Seery, D. J. and Bowman, C. T., *Combustion and Flame*, **14** (1970), 37–47.
7. Pickard, A. D. and Clark, E. R., *Talanta*, **31** (1984), 763–771.
8. Geisling, K. L., Tashima, M. K., Girman, J. R. and Miksch, R. R., *Environment International*, **8** (1982), 153–158.
9. Taylor, D. G. (Ed), *National Institute for Occupational Safety and Health Manual of Analytical Methods* (NOLSH, Cincinnati, 1977); DHHS (NIOSH) Publication No. 77-157-AP&CAM 125.
10. Möhlmann, G. R., *Applied Spectroscopy*, **39** (1) (1985), 98–101.
11. Koizumi, H. and Suzuki, Y., *Journal of Chromatography*, **333** (1988), 299–307.
12. Beasley, R. H., Hoffmann, C. E. Rueppel, M. L. and Worley, J. W., *Analytical Chemistry*, **52** (1980), 1110–1114.
13. Levin, J.-O., Lindahl, R., and Andersson, K., *Environment, Science, and Technology*, **19** (1986), 70–74.
14. Cammann, K., Faust, M., Grömping, A., Meyer, U. and Winter, B., *VDI-Berichte*, **838** (1990), 91–100.
15. Karst, U., Binding, N., Cammann, K. and Witting, U., *Verhandlungen d. Deutschen Gesellschaft für Arbeitsmedizine. V.* (1990), 327–330.
16. Grömping, A. and Cammann, K., *Fresenius Zeitschrift für Analytische Chemie*, **335** (1989), 796–801.
17. Saltzman, B. E., *Analytical Chemistry*, **26** (12) (1954), 1949–1954.
18. Sickles, J. E., Grohse, P. M., Hodson, L. L., Salmons, C. A., Cox, K. W., Turner, A. R. and Estes, E. D., *Analytical Chemistry*, **62** (1990), 338–346.
19. Kaulbach, S., *Zentralblatt gesamte Hygiene*, **35** (1989), 55–57.
20. Andersson, G., Andersson, K., Nilsson, C.-A. and Levin, J.-O., *Chemosphere*, **8** (1979), 823–827.
21. Cammann, K., Grömping, A. and Karst, U., *German Patent DPA*, No. 41 06 875, (1992).
22. Grömping, A. H. J., Karst, U. and Cammann, K., *Journal of Chromatography*, **653** (1993), 341–347 .
23. Monsen, R. M. and Stock, T. H., *Journal of Environment and Health*, **49** (2) (1986), 72–75.
24. Grömping, A. H. J. and Cammann, K., *Instrumentation Science & Technology*, **22** (1) (1994), 25–38.
25. Henke, W., Diploma thesis, Münster, Germany (1983).
26. Winter, B., Ph.D. thesis, Münster, Germany (1991).
27. Matthews, T. G. and Howell, T. C., 3rd International Conference on Indoor and Climate, Stockholm (1984).
28. Possanzini, M., Ciccioli, P., Di Palo, V. and Draisci, R., *Chromatographia*, **23** (11) (1987), 829–834.
29. Lipari, F. and Swarin, S. J., *Journal of Chromatography*, **247** (1982), 297–306.
30. Lies, K. H., *Nicht limitierte Automobil-Abgaskomponenten* (Volkswagen AG, Wolfsburg, 1988).

